# Effective use of Clozapine for challenging behaviours in neurodevelopmental disorders: A case report

**DOI:** 10.1192/j.eurpsy.2026.11252

**Published:** 2026-07-31

**Authors:** K. W. S. Lee

**Affiliations:** Developmental Psychiatry, Institute of Mental Health, Singapore, Singapore

## Abstract

**Introduction:**

Clozapine has shown potential in treating severe aggression in individuals with neurodevelopmental disorders, a population for whom pharmacological options remain limited. We present a case of effective use of Clozapine for treatment of challenging behaviours in Autism Spectrum Disorder and Intellectual Disability.

**Objectives:**

We seek to illustrate the effective use of Clozapine in an individual with Autism Spectrum Disorder and Intellectual Disability who exhibited severe aggressive and self-injurious behaviours in the psychiatric inpatient setting, along with a discussion on the key considerations and risks of the treatment that were pertinent to the case.

**Methods:**

Details of the case are described, with information gathered from past medical records.

**Results:**

The case is a 28-year-old male with a diagnosis of Autism Spectrum Disorder and Intellectual Disability. He had recurrent readmissions due to aggressive behaviour towards his mother and self-injurious behaviour (hitting his head on walls). Triggers for these episodes appeared to centre around his rigidity with demands and poor frustration tolerance.

He had been tried on multiple psychotropics in the past to manage his challenging behaviour, including Olanzapine, Trifluoperazine, Aripiprazole, Haloperidol, Chlorpromazine, Sertraline, Lithium and Carbamazepine, with limited efficacy.

He began to exhibit more severe behavioural disturbances during a prolonged hospital admission. Some of these concerning behaviours include aggression towards nursing staff and other patients, damaging of property and barging open electromagnetically-sealed doors to enter secured rooms to satisfy his demands of obtaining food or playing with electronics. There was limited efficacy with behaviour modification and alternative strategies such as providing him with activities to redirect and provide sufficient stimulation and regulation as these were limited by ward staffing resources.

The decision was made in consultation with the patient’s parents to initiate clozapine treatment in his best interests. Initial rate of up-titration of the clozapine dose was limited by side-effects of oversedation, mild tachycardia, hypersalivation and concerns for seizures given a history of generalized tonic-clonic seizures. Significant improvement in his behaviour was observed at a dose of Clozapine 250mg/day after a period of 3 months.

**Conclusions:**

Clozapine may represent a safe and effective pharmacological option for managing challenging behaviour in individuals with neurodevelopmental disorders who are unresponsive to other treatments. Further research is needed to better understand its efficacy and safety in this population.

**Disclosure of Interest:**

None Declared

